# Reimagining periodontal regeneration: smart biomaterials for a complex tissue landscape

**DOI:** 10.1093/rb/rbag182

**Published:** 2026-08-20

**Authors:** Ming Liu, Xiaobo Chen, Lili Li, Ding Wang, Yin Xiao

**Affiliations:** Xiamen Key Laboratory of Stomatological Disease Diagnosis and Treatment, Stomatological Hospital of Xiamen Medical College, Xiamen 361008, China; School of Medicine and Dentistry, Griffith University, Queensland, QLD 4222, Australia; Xiamen Key Laboratory of Stomatological Disease Diagnosis and Treatment, Stomatological Hospital of Xiamen Medical College, Xiamen 361008, China; Nanjing Stomatological Hospital, Affiliated Hospital of Medical School, Institute of Stomatology, Nanjing University, Nanjing 210000, China; Department of Obstetrics and Gynecology, Guangdong Provincial Key Laboratory of Major Obstetric Diseases, Guangdong Provincial Clinical Research Center for Obstetrics and Gynecology, The Third Affiliated Hospital, Guangzhou Medical University, Guangzhou 510150, China; School of Medicine and Dentistry, Griffith University, Queensland, QLD 4222, Australia; Institute for Biomedicine and Glycomics, Griffith University, Queensland, QLD 4222, Australia; Australian Centre for Precision Health and Technology (PRECISE), Griffith University, Queensland, QLD 4222, Australia

**Keywords:** smart biomaterials, periodontal regeneration, stimuli-responsive materials, immunomodulation, periodontal ligament–cementum–bone interface, translational research

## Abstract

Periodontitis is a prevalent chronic inflammatory disease and a major cause of tooth loss and systemic complications. Due to its progressive, often asymptomatic nature, substantial destruction of the cementum, periodontal ligament and alveolar bone is frequently present at the time of diagnosis, highlighting the urgent need for functional periodontal regeneration. Conventional biomaterials primarily serve as passive scaffolds and rarely enable the coordinated regeneration of multiple periodontal tissues. Recently, smart biomaterials have emerged as a promising strategy to address these limitations. By integrating stimulus responsiveness, bio-instructive and immunomodulatory functions and spatiotemporal control of therapeutic cues, these materials could dynamically interact with the diseased microenvironment to enhance regenerative outcomes for future periodontal therapy. In this review, we summarize the key biological and mechanistic barriers to periodontal regeneration, including multi-tissue coordination, microbial challenges, dysregulated inflammation and mechanical loading. We discuss how smart biomaterials are designed to overcome these obstacles through microenvironment-responsive delivery, immune modulation and interface-oriented regeneration. Finally, we highlight key design principles, evaluation frameworks and the translational relevance of animal models for periodontal biomaterials development. Smart biomaterials represent a transition from passive scaffolds to dynamic, immuno-instructive systems for periodontal regeneration.

## Graphical abstract

**Graphical Abstract:**
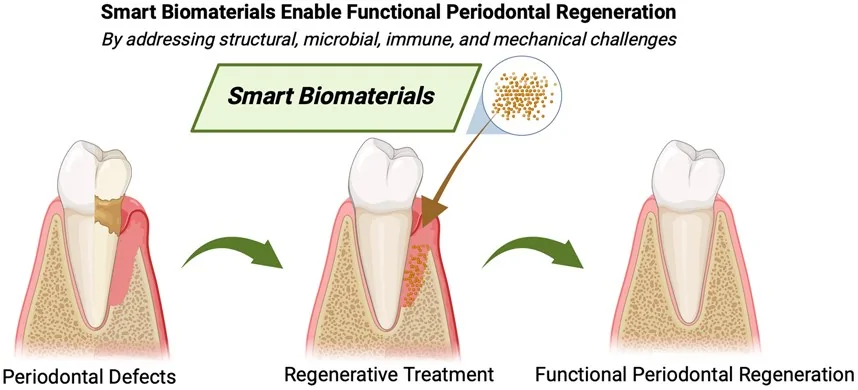

## Introduction

### Clinical significance and unmet needs in periodontal regeneration

Chronic periodontitis remains a widespread inflammatory disease, impacting up to 50% of adults globally and causing progressive deterioration of the tooth-attachment apparatus, namely the gingiva, alveolar bone, cementum and periodontal ligament [1]. If unmanaged, this condition not only leads to tooth loss but is also linked to broader health issues such as cardiovascular conditions, diabetes and pregnancy complications [2]. Standard treatments, including nonsurgical and surgical debridement techniques, as well as regenerative procedures such as guided tissue regeneration (GTR), aim to control microbial infection, resolve inflammation and guide tissue regeneration. However, these approaches tend to yield partial repair rather than proper regeneration of the periodontium [3].

A central clinical challenge lies in the need to simultaneously and coordinately regenerate three highly specialized yet interdependent tissues, such as cementum, PDL and alveolar bone, each possessing distinct cellular compositions, extracellular matrices and biomechanical roles (Figure 1). This challenge is particularly pronounced in complex periodontal defects, such as deep intrabony defects, Class II/III furcation involvements and combined periodontal lesions, which are characterized by irregular anatomical configurations and compromised regenerative microenvironments [4, 5]. Successful regeneration requires not only the formation of these tissues individually but also the precise re-establishment of their spatial alignment and structural continuity, particularly the insertion of well-oriented Sharpey’s fibers into newly formed cementum and bone to restore functional load transmission [6]. Achieving such spatiotemporal coordination within the narrow periodontal space, where competing epithelial and connective tissue cells migrate rapidly, remains a major obstacle in current regenerative strategies.

**Figure 1:**
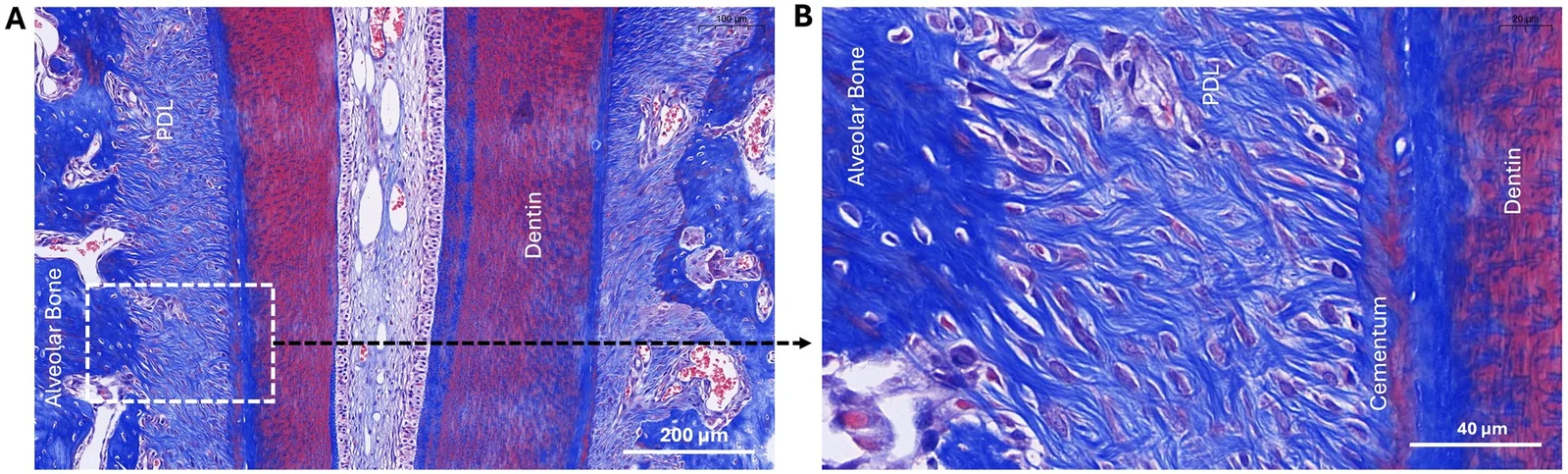
Histological structure of periodontal tissues. Representative images show the organization of alveolar bone, periodontal ligament (PDL) and cementum. (**A**) Low magnification (100×) reveals the spatial relationships among the alveolar bone, PDL, cementum and dentin. (**B**) Higher magnification (400×) clearly shows PDL fibers inserting into alveolar bone and cementum. Scale bars: A, 200 μm; B, 40 μm.

Another critical barrier is the persistent challenge of dental plaque control during the regeneration process. The periodontal wound environment is continuously exposed to a complex and dynamic biofilm, which can rapidly recolonize the root surface and biomaterial interfaces, sustaining inflammation and disrupting cell recruitment, differentiation and matrix maturation. Inadequate control of microbial challenges during early healing can compromise clot stability, impair attachment formation and ultimately shift healing toward long junctional epithelium rather than true periodontal regeneration [7, 8].

These biological and clinical complexities are further aggravated by systemic conditions such as diabetes, smoking and aging, which impair angiogenesis, stem/progenitor cell function and immune resolution, thereby reducing regenerative predictability. Consequently, despite decades of research, no existing therapy consistently succeeds in restoring full function and architecture [9].

Driven by this substantial unmet need, research has increasingly focused on multifunctional smart biomaterials capable of orchestrating cell behavior, modulating inflammation and resisting microbial colonization while providing spatial and mechanical cues for tissue-specific regeneration. The convergence of biomaterials science, stem cell biology and tissue engineering thus offers a potential framework to move beyond passive scaffolding toward biologically instructive platforms that actively coordinate structural reconstruction and host–microbe balance in periodontal healing.

### Emergence of smart materials in periodontal tissue engineering

Biomaterials have long been considered essential components of periodontal regenerative therapies, primarily serving as structural scaffolds to support tissue ingrowth and space maintenance [10]. Early-generation materials, such as nonresorbable membranes and inert grafts, are designed to play a passive role, simply separating tissues, maintaining wound stability or preventing epithelial downgrowth. While these materials have provided modest improvements in bone fill and clinical attachment levels, their limited bioactivity and lack of tissue specificity have limited their long-term effectiveness and clinical predictability [11].

In recent decades, the understanding of tissue regeneration has shifted from a purely mechanical paradigm toward a more biological and dynamic framework [12]. This has redefined the role of biomaterials: rather than being passive carriers, advanced materials are now expected to engage actively with the surrounding biological environment, modulate cellular behavior and orchestrate tissue-specific healing [12]. The bio-instructive platforms are designed to interact with host cells and molecules, deliver growth factors in a controlled manner, promote angiogenesis, modulate immune responses and even direct stem cell differentiation [13]. As such, the evolution of biomaterials represents more than just improvements in material composition, which reflects a fundamental transition in regenerative strategy. The focus has shifted from ‘filling the defect’ to ‘regenerating the system’, with biomaterials functioning as intelligent mediators that bridge the gap between biology and engineering [14]. This transformation underscores the growing importance of biomaterial design in driving next-generation periodontal regenerative solutions.

### Scope and objectives of this review

Several recent reviews have summarized the material composition, scaffold fabrication strategies and biological applications of biomaterials for periodontal regeneration [15–18]. However, most existing reviews focus primarily on material classification, growth factor delivery or general tissue engineering approaches, with limited emphasis on how biomaterials dynamically interact with the evolving periodontal microenvironment amid inflammatory, microbial and mechanical challenges.

This review provides a distinct perspective by framing smart biomaterials not simply as structural scaffolds but as dynamic, immuno-instructive and mechanoresponsive therapeutic systems. We critically integrate recent advances in microenvironment-responsive delivery, immune modulation, bioelectronic/mechanical adaptation and translational validation, while explicitly identifying current knowledge gaps and barriers to clinical implementation. By linking material design principles with biological complexity and translational readiness, this review aims to provide a more clinically relevant framework for the next generation of periodontal regenerative biomaterials.

Specifically, this review is organized around stimuli-responsive, bioinstructive, immunomodulatory and bioelectronic/mechanoresponsive biomaterial systems and critically evaluates their biological mechanisms, regenerative performance and translational readiness.

### Literature search strategy

A structured literature search was conducted using electronic databases, including PubMed, Scopus and Web of Science, to identify relevant studies, with the final search performed on 15 May 2026. The search strategy combined terms related to periodontal regeneration and smart biomaterials: (‘periodontal regeneration’ OR ‘periodontal tissue regeneration’ OR ‘periodontal defect’) AND (‘smart biomaterial’ OR ‘stimuli-responsive’ OR ‘responsive biomaterial*’ OR ‘biomimetic scaffold*’ OR hydrogel* OR ‘immunomodulatory biomaterial*’ OR ‘electroactive scaffold*’ OR ‘piezoelectric scaffold*’ OR ‘conductive scaffold*’).

The initial search identified 928 records (PubMed, *n* = 205; Scopus, *n* = 281; Web of Science, *n* = 442). After duplicate removal, 599 records remained for title and abstract screening. Following title and abstract screening, 445 studies were excluded, including studies unrelated to periodontal regeneration (*n* = 203), non-English publications (*n* = 12), conference abstracts or records without sufficient full data (*n* = 47) and studies lacking mechanistic or translational relevance (*n* = 183). The remaining 154 studies underwent full-text assessment and were considered for the final evidence synthesis.

## Smart materials in the context of periodontal regeneration

### Concept of smart biomaterials

Smart biomaterials are a class of functional materials engineered to interact dynamically with the biological environment. Unlike traditional biomaterials that serve mainly as passive structural supports, smart biomaterials possess the capacity to sense, respond to and even adapt to local biochemical or biophysical cues, thereby playing an active role in directing tissue regeneration [19]. These materials are designed to mimic or influence physiological processes in a controlled, often stimulus-triggered manner, aligning with the principles of regenerative medicine, precision therapy and systems biology.

### Classification of smart biomaterials

Based on their functional roles and mechanisms of action, smart biomaterials can be classified into several distinct, often overlapping categories. Each class employs specific strategies, whether chemical, mechanical, biological or computational, to interact with the host environment, respond to dynamic cues and drive targeted regenerative processes. Based on their primary functions, smart biomaterials for periodontal regeneration can be broadly categorized into four overlapping groups:

#### Stimuli-responsive materials

Stimuli-responsive biomaterials have emerged as an important strategy for periodontal regeneration because they can dynamically respond to pathological changes within the periodontal microenvironment. These respond to specific environmental triggers such as temperature (thermo-responsive), pH (pH-responsive), enzymes (enzyme-responsive), redox conditions (redox-responsive), light (photo-responsive) or magnetic/electric fields. Their responsiveness can be leveraged to control the release of bioactive agents, scaffold degradation or conformational changes at the site of implantation [20]. In this section, we summarize representative stimuli-responsive systems according to different triggering mechanisms and discuss their regenerative applications (Figure 2).

**Figure 2:**
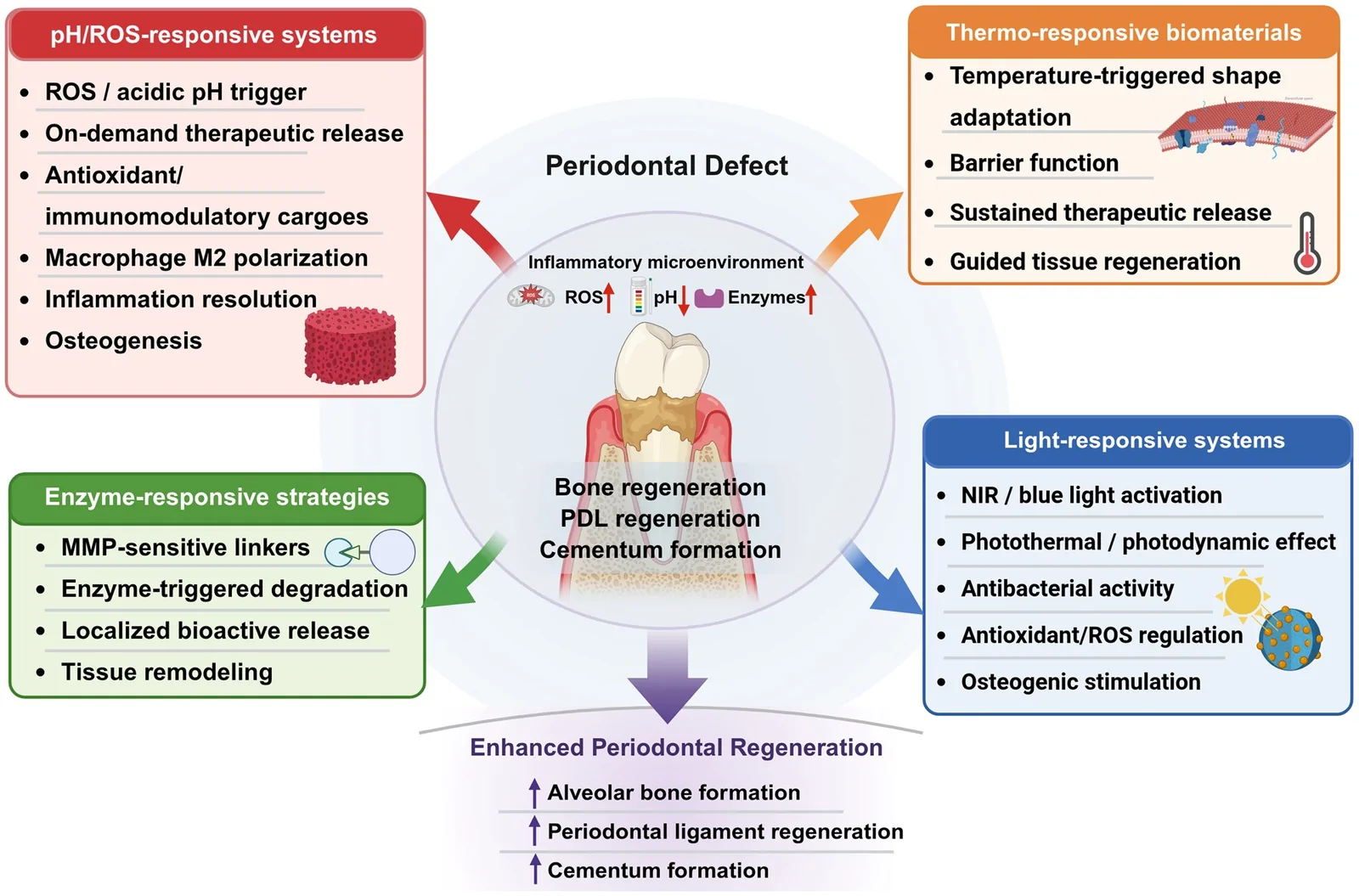
Stimuli-responsive smart biomaterials for periodontal regeneration.

#### pH- and redox-responsive biomaterials

In the inflammatory microenvironment of chronic periodontitis, elevated levels of reactive oxygen species (ROS) and acidic pH are hallmarks [21]. To exploit these cues, pH- and ROS-responsive platforms have demonstrated the ability to achieve on-demand release of therapeutic agents. Representative pH/ROS-responsive systems have incorporated antioxidant or immunomodulatory agents such as Epigallocatechin-3-gallate [22] and anti-inflammatory nanoparticles [23], enabling microenvironment-triggered release in response to oxidative stress and pH fluctuations. These platforms have been shown to attenuate local inflammation, respond to excessive ROS, promote macrophage polarization toward a pro-regenerative phenotype and enhance alveolar bone regeneration [24, 25]. Beyond regulating drug release, pH- and redox-responsive chemistries have also been utilized to control hydrogel formation, crosslinking density, stiffness and degradation behavior [26, 27]. Such dynamic networks can undergo localized gelation within inflammatory periodontal defects while continuously adapting their mechanical properties and degradation rates to changes in the pathological microenvironment, thereby improving defect conformity and therapeutic performance [28].

#### Thermo-responsive biomaterials

##### Thermo-responsive membranes

Thermo-responsive membranes have emerged as promising alternatives to conventional GTR membranes by integrating temperature-dependent shape adaptability, defect conformity and structural stability under physiological conditions [29]. Through temperature-triggered shape transformation or phase transition, thermo-responsive biomaterials can dynamically adapt to defect geometry during implantation while maintaining structural integrity after placement [29, 30]. Such behavior improves defect coverage, facilitates space maintenance and enhances the regenerative microenvironment required for periodontal tissue repair.

Representative thermo-responsive membranes have been fabricated using combinations of biodegradable polymers such as poly(L-lactic acid) (PLLA), poly(ε-caprolactone) diacrylate (PCL-DA) and poly(lactide-co-glycolide) diacrylate (PLGA-DA), enabling temperature-dependent shape plasticity together with mechanical rigidity at physiological temperature [29, 31]. Through thermally induced phase separation and self-assembly, these membranes can generate biphasic architectures composed of dense barrier layers that prevent undesirable soft-tissue invasion and porous or nanofibrous compartments that facilitate cell infiltration and tissue regeneration [32, 33], thereby creating a favorable microenvironment for periodontal regeneration. Furthermore, incorporation of PLGA-based components can regulate membrane degradation behavior and facilitate therapeutic loading and controlled release, thereby supporting scaffold remodeling and tissue regeneration [34, 35].

##### Thermosensitive hydrogels

While thermo-responsive membranes primarily improve defect coverage and space maintenance, thermosensitive hydrogels provide additional advantages through minimally invasive delivery and *in situ* gelation [20, 36]. These systems can be injected into liquid precursors and subsequently undergo temperature-triggered gelation within periodontal defects, enabling precise filling of irregular anatomical structures and prolonged retention of therapeutic agents.

Many thermosensitive hydrogels further exhibit shear-thinning, self-healing and shape-conforming properties, facilitating clinical handling while maintaining intimate contact with defect surfaces under dynamic oral conditions [20, 36, 37]. Commonly employed polymers include poloxamers, poly(N-isopropylacrylamide) (PNIPAM) and chitosan derivatives, which undergo sol–gel transition near physiological temperature [36, 38]. This temperature-triggered gelation enables the formation of stable hydrogel networks capable of sustained therapeutic release while maintaining structural integrity under mechanical stresses in the oral environment [39, 40].

Beyond improving local drug retention, thermosensitive hydrogels have demonstrated the ability to enhance alveolar bone regeneration, periodontal ligament formation and overall periodontal healing through localized and sustained delivery of growth factors, extracellular vesicles, antimicrobial agents and immunomodulatory cargos [15, 20, 41]. By improving local retention, protecting bioactive agents from rapid degradation and enabling controlled release within periodontal defects, these thermogelling systems can create a more favorable microenvironment for tissue regeneration and functional periodontal repair [42, 43].

##### Enzyme-responsive biomaterials

Enzyme-responsive biomaterials are engineered to undergo degradation, structural remodeling or therapeutic activation in response to disease-associated enzymes that are overexpressed within pathological microenvironments, particularly matrix metalloproteinases (MMPs), hyaluronidases and cathepsins [44, 45]. By incorporating enzyme-cleavable linkers, peptide substrates or degradable crosslinking motifs, these systems can achieve site-specific degradation and on-demand release of therapeutic cargos in response to local enzymatic activity [45, 46]. Such enzyme-triggered activation enables spatiotemporally controlled therapeutic delivery while minimizing off-target release and improving treatment precision within pathological tissues [47].

Beyond controlled drug delivery, enzyme-responsive mechanisms can also regulate scaffold remodeling and degradation kinetics, allowing biomaterials to dynamically adapt to the evolving tissue healing process [48, 49]. Such spatiotemporally regulated degradation has been shown to improve cellular infiltration, extracellular matrix deposition, vascularization and tissue regeneration while minimizing premature loss of structural support [50, 51]. Owing to their ability to couple therapeutic activation with pathological enzyme expression, enzyme-responsive biomaterials represent a promising strategy for achieving precise and adaptive regenerative therapies in inflammatory tissue defects [52].

##### Light-responsive biomaterials

Although light-responsive materials are still emerging in the context of periodontal regeneration, they offer spatially and temporally precise control over biomaterial behavior, such as drug release or degradation. A near-infrared (NIR)-responsive copper-cerium oxide nanozyme showed strong antibacterial, antioxidant and osteogenic effects under 808 nm irradiation by modulating the Nrf2/HO-1 pathway [53]. A blue light-activated hydrogel loaded with *Lactobacillus rhamnosus* GG provided sustained antibacterial and immunomodulatory effects, promoting periodontal regeneration [54]. CeVO₄/Ag nanohybrids and gold-coated magnetic nanoparticles also demonstrated efficient NIR-triggered photothermal and photodynamic activity, highlighting their potential for light-guided periodontal treatment [55, 56].

Although stimuli-responsive systems have shown promising therapeutic potential in periodontal regeneration, several important limitations remain. First, most currently reported systems have been validated only in rodent models, with limited evidence from large-animal studies or clinically relevant defect models. Second, while pH-, ROS- and enzyme-responsive platforms consistently demonstrate improved local drug delivery and reduced inflammatory burden, direct comparisons between different trigger mechanisms remain difficult due to heterogeneity in scaffold composition, defect models and outcome measures. Furthermore, most studies focus primarily on alveolar bone regeneration, whereas evidence for predictable cementum regeneration and functional Sharpey’s fiber insertion remains limited. These findings suggest that future studies should move beyond proof-of-concept designs toward standardized comparative studies and long-term functional validation.

#### Bioinstructive materials

These materials are engineered to deliver biological signals that actively guide cellular behaviors such as adhesion, proliferation, differentiation and migration. This may involve surface modification, incorporation of specific ligands or presentation of matrix-mimetic features that mimic native tissue environments [57, 58]. The following sections summarize the major approaches used to guide tissue-specific periodontal regeneration (Figure 3).

**Figure 3:**
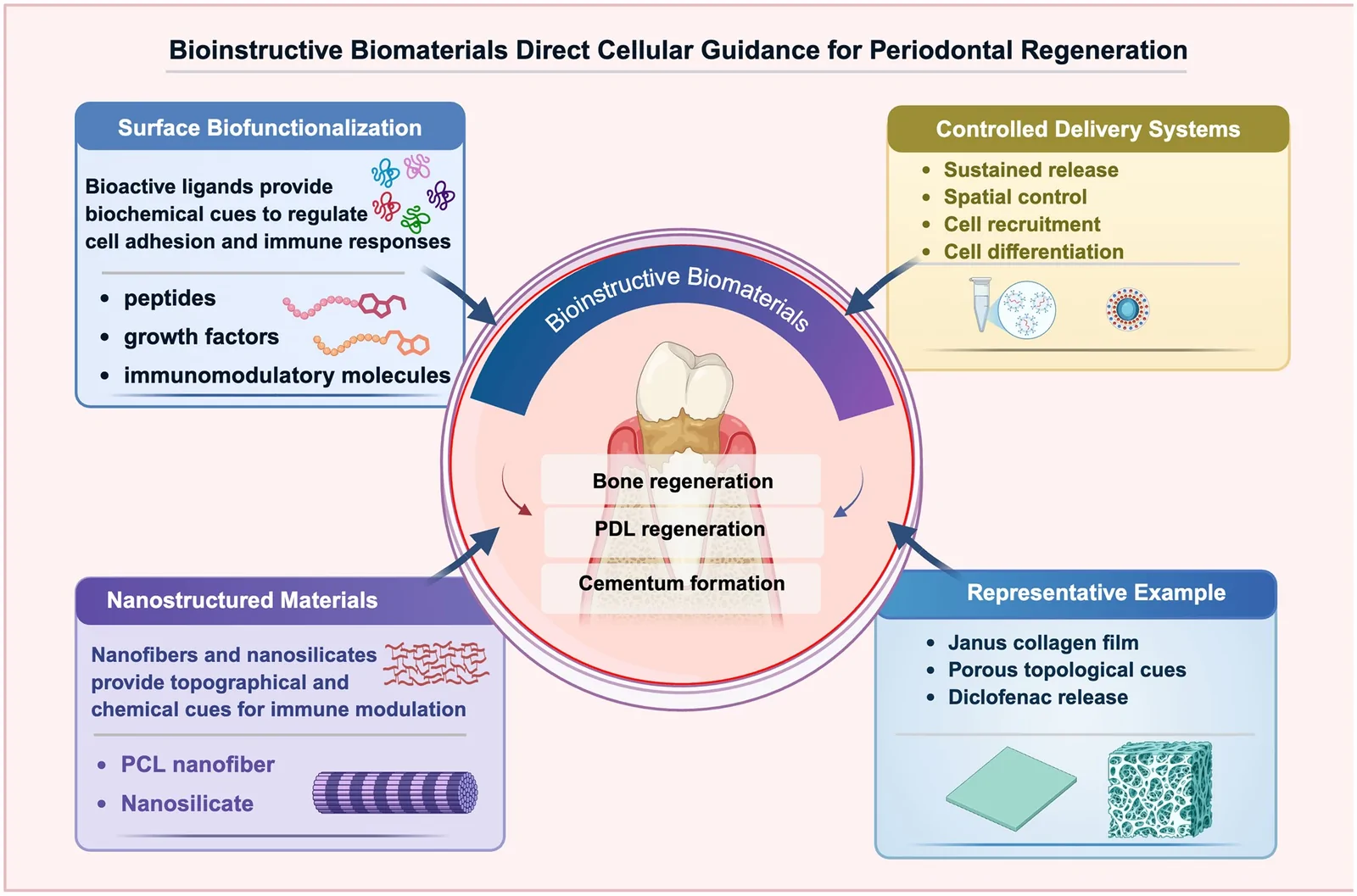
Bioinstructive biomaterials direct cellular guidance and tissue-specific regeneration in periodontal defects.

### Surface modification and ligand functionalization

Modern scaffold engineering emphasizes the surface modification of biomaterials to impart biological signaling capabilities. Functionalization with bioactive ligands, such as peptides, growth factors and immunomodulatory molecules, enhances cellular adhesion, migration and immune crosstalk. For example, Lei *et al.* [28] developed a programmable Janus collagen film with a porous, drug-loaded surface layer via electro-assembly. The combination of porous topological cues and sustained diclofenac sodium release promoted macrophage polarization toward a pro-healing phenotype and enhanced osteogenic differentiation, illustrating a dual bioinstructive effect.

### Nanostructured materials

Nanostructured biomaterials provide topographical and physicochemical cues that influence cellular behavior at the microscale and nanoscale. Nanofibrous architectures can mimic the native extracellular matrix, thereby facilitating cell attachment, alignment and differentiation. For example, nanosilicate-laden PCL nanofibrous membranes developed by Xu *et al.* [59] provided both topographical and chemical cues to periodontal ligament cells (PDLCs), promoting osteogenic differentiation, demonstrating the potential of nanostructured scaffold design for periodontal regeneration.

### Controlled delivery of bioactive signals

Beyond surface engineering, smart biomaterials can function as delivery platforms for growth factors, exosomes, therapeutic drugs and bioactive nanoparticles. Through sustained, spatially controlled release, these systems provide continuous biological instruction to surrounding cells, thereby enhancing recruitment, differentiation and tissue regeneration. By integrating structural support with dynamic signaling functions, controlled delivery systems further expand the regenerative potential of bioinstructive biomaterials.

Although bioinstructive biomaterials have demonstrated a promising ability to guide cell behavior and tissue-specific regeneration, current evidence remains largely limited to preclinical studies. In addition, the relative contribution of surface biofunctionalization, nanotopographical cues and controlled delivery mechanisms is often difficult to distinguish, making cross-study comparisons challenging. Furthermore, the predictable regeneration of functional cementum and Sharpey’s fiber insertion remains insufficiently demonstrated.

### Immunomodulatory materials

A growing subclass of smart biomaterials is designed to modulate host immune responses, such as promoting anti-inflammatory macrophage phenotypes (M2 polarization), recruiting regulatory T cells, as well as directing neutrophil-mediated immune regulation [60]. These properties are especially relevant in periodontal applications, where inflammation is a major barrier to successful regeneration [61]. Because persistent inflammation remains a major obstacle to periodontal regeneration, increasing attention has been directed toward immunomodulatory biomaterials that reshape the local immune microenvironment. Representative strategies are discussed below (Figure 4).

**Figure 4:**
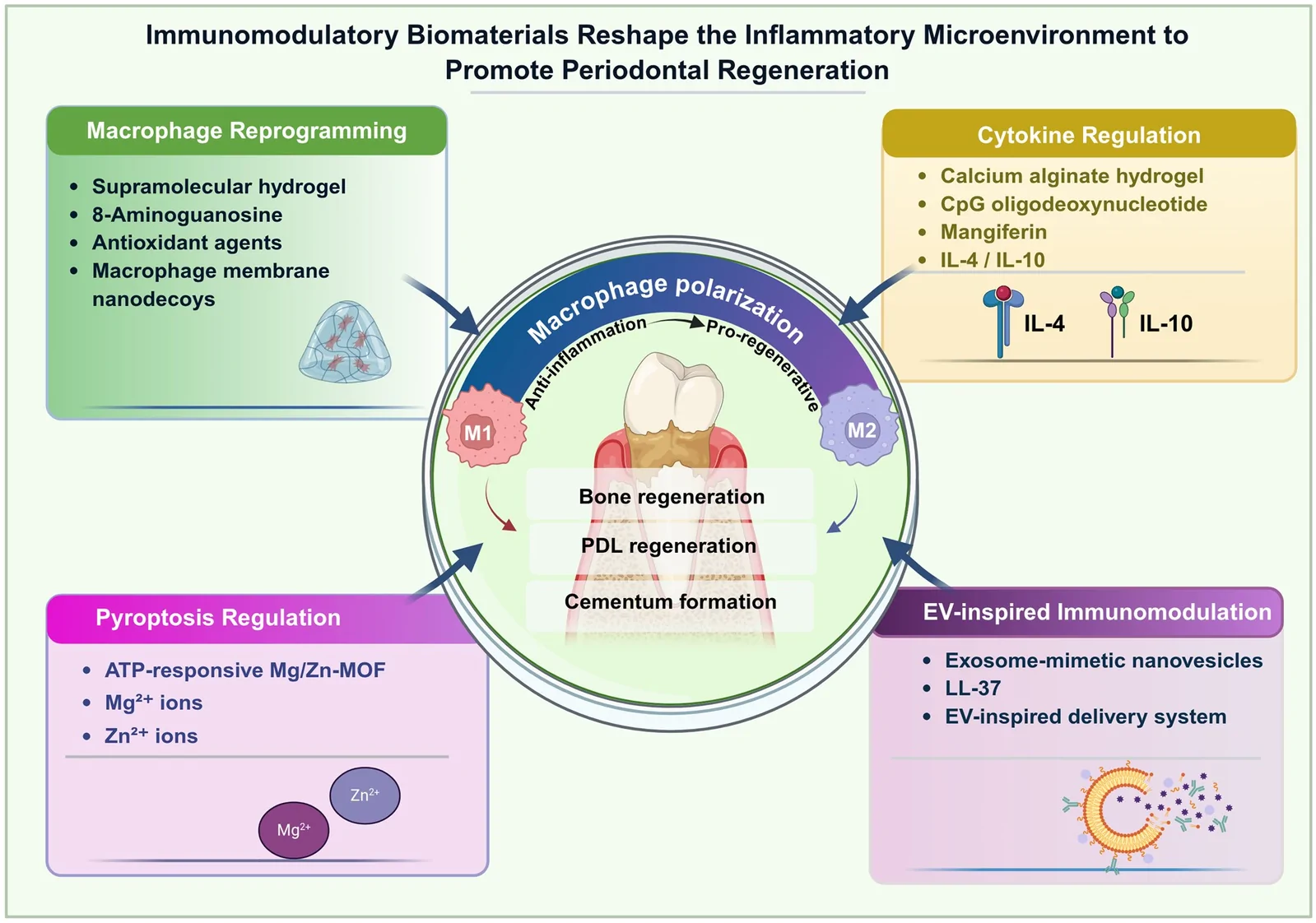
Immunomodulatory biomaterials reshape the inflammatory microenvironment to promote periodontal regeneration.

### Immunomodulation via macrophage polarization

Immuno-instructive biomaterials specifically target the immune system’s behavior. The periodontal immune microenvironment comprises multiple innate and adaptive immune cell populations that collectively regulate inflammation and tissue regeneration [62, 63]. Neutrophils serve as the first line of host defense by controlling microbial invasion, although their persistent activation can contribute to excessive tissue destruction through the release of reactive oxygen species, proteases and neutrophil extracellular traps (NETs) [64, 65]. Adaptive immune cells, particularly T cells, also play important roles in periodontal healing. Regulatory T cells (Tregs) promote inflammation resolution and tissue repair, whereas Th17 cells are associated with sustained inflammation and osteoclast-mediated bone resorption [62, 66]. Consequently, modulating the interactions among these immune cell populations has emerged as an important strategy for promoting a pro-regenerative periodontal microenvironment.

Among these immune cells, macrophages remain the most extensively investigated therapeutic target for smart biomaterials because of their remarkable phenotypic plasticity and central role in orchestrating both inflammatory resolution and tissue regeneration [67–69]. One of the key strategies is directing macrophage polarization from a pro-inflammatory M1 phenotype toward a regenerative M2 phenotype, a shift that plays a critical role in periodontal tissue healing [70, 71]. Several biomaterial platforms have been developed to directly reprogram macrophage phenotypes. Shuai *et al.* [72] introduced a supramolecular hydrogel that self-delivers 8-aminoguanosine (8AG) and antioxidant agents. The hydrogel reduced M1 macrophage polarization, suppressed inflammatory responses and significantly improved periodontal healing *in vivo*. Lin *et al.* [73] took a more biologically biomimetic approach by cloaking nanodecoys with engineered macrophage membranes enriched with toll-like receptors and antimicrobial peptides (LL-37). These nanodecoys actively reshaped the inflammatory microenvironment through targeted bacterial elimination and NRF2 pathway activation, which in turn promoted M2 macrophage polarization and osteogenic differentiation. The strategy led to enhanced alveolar bone regeneration in a periodontitis model.

### Cytokine-mediated immune regulation

Beyond direct macrophage reprogramming, biomaterials can also regulate the immune microenvironment by controlling cytokine release and signaling. A study by Gu *et al.* [74] demonstrated that calcium alginate hydrogels embedded with CpG ODN and mangiferin (MAG) enhanced local release of IL-10 and IL-4, suppressing osteoclastogenesis and inflammatory signaling. The combinatorial delivery attenuated RANKL-mediated bone resorption and supported alveolar bone regeneration, validating the therapeutic value of cytokine-based immune modulation.

### Regulation of pyroptosis and inflammatory cell death

In addition to cytokine modulation, emerging biomaterials have been designed to target inflammatory cell death pathways associated with periodontal tissue destruction. Yang *et al.* [75] explored a metal–organic framework (Mg/Zn-MOF) that responded to extracellular ATP levels typical of periodontitis sites. Upon activation, the scaffold released immunomodulatory ions that suppressed pyroptosis via the Caspase-1/GSDMD and Caspase-11 pathways, preserving collagen and preventing further bone degradation.

### Extracellular vesicle-inspired immunomodulation

Extracellular vesicle-inspired platforms represent another promising strategy for immune regulation and periodontal regeneration. A delivery system incorporating exosome-mimetic nanovesicles and antimicrobial peptides achieved simultaneous bacterial clearance and immune modulation through macrophage reprogramming [73], thereby promoting periodontal regeneration.

Collectively, these studies demonstrate that immunomodulatory biomaterials can regulate multiple components of the host immune response, ranging from macrophage polarization and cytokine secretion to pyroptosis inhibition and extracellular vesicle-mediated signaling.

Although macrophage-targeted immunomodulation has emerged as a central strategy in periodontal regeneration, current evidence is largely derived from short-term rodent studies. While many platforms consistently report enhanced M2 polarization and improved osteogenesis, the durability of immune reprogramming remains unclear. In addition, most studies rely on simplified M1/M2 classification, which may not fully reflect the complexity of immune responses in chronic periodontitis. Whether these immunomodulatory effects can be maintained under long-term microbial challenge and functional loading remains unknown.

### Bioelectronic and mechanoresponsive materials

Mechanical stimulation is essential for functional periodontal regeneration. Accordingly, bioelectronic and mechanoresponsive biomaterials have been developed to integrate mechanical cues with regenerative signaling. These materials are designed to sense, generate or transmit electrical and mechanical signals that regulate cellular behavior and tissue regeneration. Examples include piezoelectric materials, conductive polymers and mechanically active scaffolds that convert physical stimuli into bioelectrical cues or activate mechanotransduction pathways, thereby promoting cell proliferation, differentiation and functional tissue integration [76, 77]. Representative approaches are summarized below (Figure 5).

**Figure 5:**
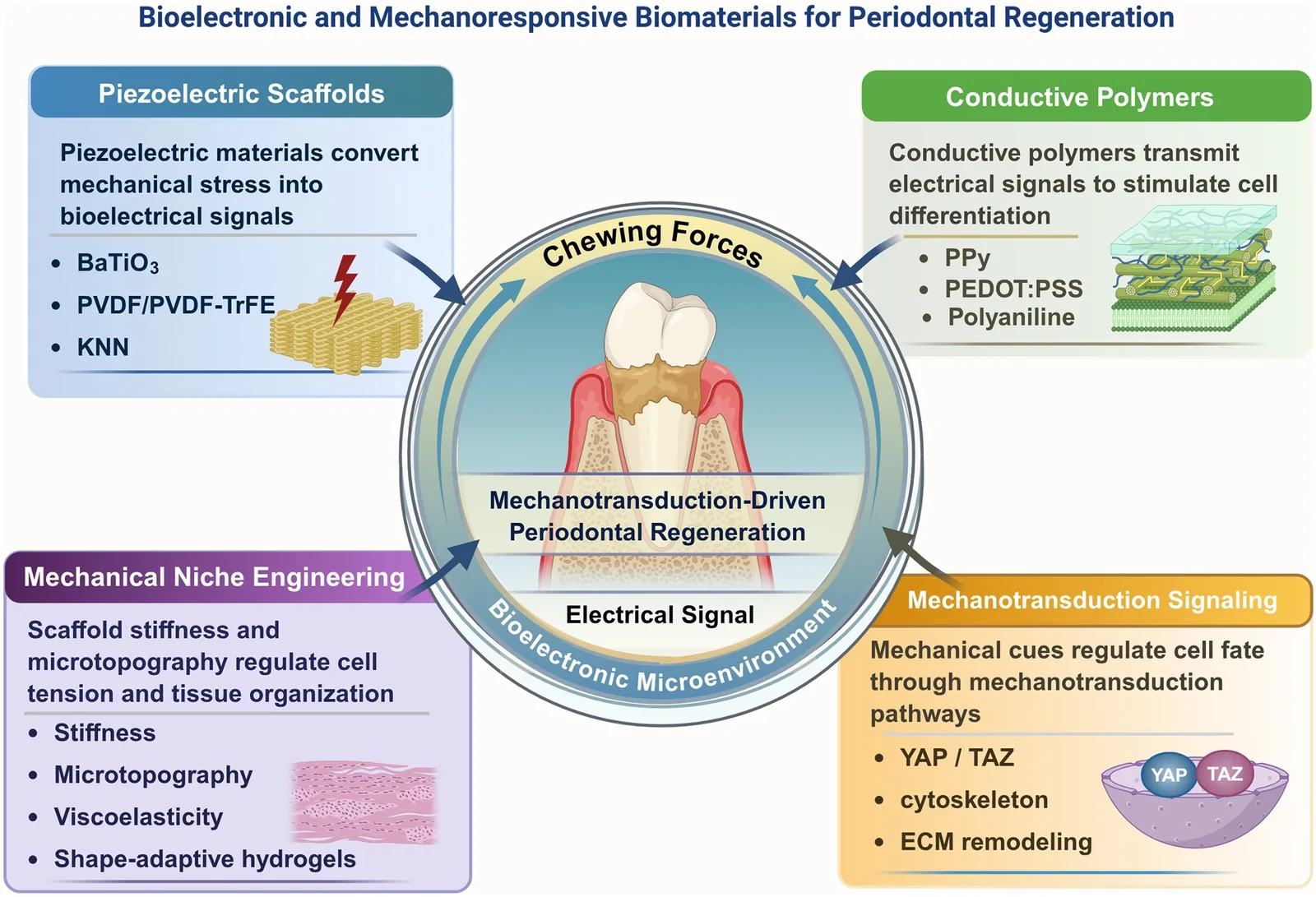
Bioelectronic and mechanoresponsive biomaterials for periodontal regeneration.

### Piezoelectric and conductive biomaterials

Piezoelectric materials such as barium titanate (BaTiO₃) and polyvinylidene fluoride (PVDF) have demonstrated significant potential for mimicking the electromechanical microenvironment of periodontal tissues [78]. For example, chitosan membranes incorporating 3% and 6% BaTiO₃ nanoparticles exhibited improved hydrophilicity, enhanced antibacterial activity and higher surface roughness and zeta potential, as demonstrated by Houshyar *et al.* [79], which are conducive to cell adhesion and differentiation. These membranes also generated piezoelectric currents under mechanical stimulation, suggesting a capacity to activate downstream signaling pathways involved in tissue repair.

Similarly, PVDF-TrFE nanofibrous scaffolds have been shown to boost osteogenic gene expression (Runx2 and OPN) and promote bone formation *in vivo*, particularly under cyclic mechanical loading [80]. Piezoelectricity thus serves as a built-in stimulation mechanism that does not rely on external power sources. In addition, materials like KNN (potassium sodium niobate) incorporated into polymeric scaffolds have been explored for their eco-friendly piezoelectric performance and shown to induce osteogenic differentiation in stem cells without supplementary biochemical factors [81].

Conductive polymers, including polypyrrole (PPy), PEDOT: PSS and polyaniline, also facilitate electrically mediated regeneration [82, 83]. These polymers can act as transducers or direct current carriers. One study showed that 3D-printed PLA scaffolds coated with PPy supported PDLSC proliferation and osteogenesis without electrical preconditioning [84]. Another work demonstrated that electrically conductive PEDOT hydrogels enhanced the release of endogenous growth factors from host cells and triggered osteoangiogenic pathways [85].

More recently, electroactive hydrogels incorporating piezoelectric nanoparticles have emerged as a promising alternative to conventional piezoelectric membranes and fibrous scaffolds [86]. By embedding piezoelectric nanoparticles such as BaTiO₃, ZnO or KNN within injectable hydrogel matrices, these systems combine the advantages of defect-conforming delivery, soft-tissue compatibility and mechanoelectrical stimulation [87–89]. Under physiological loading, the embedded nanoparticles can convert mechanical forces into localized electrical signals, potentially enhancing osteogenesis, angiogenesis and periodontal ligament regeneration [90]. Compared with many conventional hydrogels discussed in this review, piezoelectric nanocomposite hydrogels may additionally provide self-powered bioelectrical stimulation while maintaining favorable mechanical adaptability to irregular periodontal defects (Supplementary Table S1) [86, 91]. Nevertheless, further optimization of nanoparticle dispersion, electrical output and biomechanical compatibility remains necessary for clinical translation [90, 92].

Beyond piezoelectric biomaterials, magnetoelectric materials have recently emerged as another promising platform for tissue engineering applications. These materials couple magnetic and electrical responses, enabling the generation of localized electrical stimulation under externally applied magnetic fields [93]. Compared with conventional electrical stimulation systems, magnetoelectric biomaterials offer the advantage of wireless and noninvasive activation, allowing spatiotemporal regulation of cellular behavior without implanted electrodes [94]. Recent studies have demonstrated their ability to promote osteogenic differentiation, enhance cell proliferation and modulate tissue regeneration through electrically mediated signaling pathways [95–97]. Although their application in periodontal regeneration remains largely unexplored, the unique capability of remotely controlled bioelectrical stimulation suggests considerable potential for future periodontal tissue engineering strategies.

### Mechanotransduction-based periodontal regeneration stimulation

Mechanical loading is a natural part of the periodontal environment and plays a central role in maintaining homeostasis and triggering regeneration. Recent single-cell transcriptomic studies have identified mechanoresponsive subtypes within PDL fibroblasts, marked by the expression of ECM-remodeling genes under tensile or compressive strain [98]. A key transcriptional regulator, JDP2, integrates mechanosensitive signals and controls matrix remodeling, a critical process in early regeneration [99].

At the material interface, scaffold geometry and microtopography modulate intracellular tension through YAP/TAZ signaling, a key effector of mechanical signal transduction, as demonstrated by the differential YAP/TAZ activation observed in response to scaffold pore curvature [100]. Aligned or micropatterned scaffolds promoted nuclear translocation of YAP, cytoskeletal rearrangement and expression of osteogenic markers in PDLSCs [76]. These topographic cues were also shown to facilitate organized ligament-like structure regeneration when implanted *in vivo* [76, 101]. Additionally, hydrogel stiffness and viscoelasticity have been engineered to optimize the mechanical niche for periodontal stem cell behavior, further reinforcing the idea that mechanical context is as critical as biochemical signals [102].

Beyond electrical stimulation, the mechanical properties of biomaterials can also influence regenerative outcomes. Shape-adaptive hydrogels with shear-thinning behavior can flow under injection pressure while maintaining structural integrity after placement. Such rheological tunability improves adaptation to irregular defects and provides a mechanically favorable microenvironment for tissue regeneration.

Despite these promising findings, evidence for bioelectronic and mechanoresponsive biomaterials remains largely preclinical. Direct comparison across studies is difficult because material composition, electrical output, mechanical properties, stimulation conditions and defect models vary substantially. In addition, the long-term safety, durability and functional relevance of electrically or mechanically mediated periodontal regeneration remain insufficiently established, particularly under sustained microbial challenge and occlusal loading.

## Mechanistic integration of smart biomaterials in periodontal regeneration

The periodontium comprises the alveolar bone, PDL, cementum and gingiva, all of which require coordinated regeneration within a complex, microbially challenged environment. Smart biomaterials can address these challenges through multifunctional and spatiotemporally controlled regenerative strategies.

### How smart biomaterials work

These materials can synchronize tissue-specific cues through controlled spatial and temporal delivery of bioactive factors, including growth factors, chemokines and immunomodulatory agents [20]. They can also modulate cellular behavior by providing dynamic mechanical and topographical stimuli, promoting directed stem cell differentiation and guided tissue remodeling [103, 104]. In particular, the incorporation of angiogenic and osteoinductive molecules within smart scaffolds facilitates neovascularization and bone regeneration [105], which are essential for the survival and integration of newly formed periodontal tissues.

### Immunomodulation is a key feature

Smart biomaterials often possess immuno-instructive properties, enabling them to interact with immune cells and direct inflammation toward a pro-regenerative phenotype. By guiding macrophage polarization from pro-inflammatory M1 to anti-inflammatory M2 states, these materials help establish a regenerative niche that favors tissue integration rather than fibrotic encapsulation or chronic inflammation [106, 107].

### Current achievements

Consistent with these mechanisms, accumulating evidence demonstrates that spatially graded scaffolds, controlled growth factor delivery systems, immunomodulatory biomaterials and angiogenic platforms can promote compartment-specific regeneration of alveolar bone and PDL, enhance neovascularization and facilitate favorable immune responses (Table 1). For example, biphasic scaffolds containing osteoinductive and fibroconductive domains have been shown to support simultaneous regeneration of bone and ligament tissues [115], while immunomodulatory hydrogels can suppress local inflammation while delivering tissue-specific bioactive signals [116].

**Table 1:** Current evidence and knowledge gaps in smart biomaterial-based periodontal regeneration.

Aspect	What is established	What remains unclear
Spatial guidance	Biphasic/multiphasic scaffolds can differentially support bone and PDL formation [108, 109]	How to reproducibly induce true cementum regeneration and Sharpey’s fiber insertion in humans
Growth factor delivery	Controlled release improves osteogenesis and angiogenesis [110]	Optimal combinations, timing and doses for coordinated cementogenesis–ligamentogenesis
Immunomodulation	Macrophage M2 polarization enhances regenerative microenvironment [111]	Long-term immune reprogramming and interaction with adaptive immunity
Mechanical cues	Aligned fibers and stiffness gradients guide PDL cell orientation [109, 112]	How to dynamically adapt mechanics during healing and functional loading
Antimicrobial function	Antibacterial coatings and drug-releasing scaffolds reduce early biofilm [111]	How to maintain long-term plaque control without impairing cell attachment
Vascularization	VEGF-incorporated scaffolds enhance neovascularization [113, 114]	Integration of vascular, neural and immune networks simultaneously

### Future perspective for the integration of smart biomaterials in periodontal regeneration

Periodontal regeneration should not be regarded simply as the replacement of lost alveolar bone but as the coordinated reconstruction of a hierarchically organized cementum–periodontal ligament–alveolar bone complex within a microbially contaminated, chronically inflamed, mechanically active and anatomically irregular environment. From this perspective, the value of smart biomaterials lies not merely in their individual responsive or bioactive properties but in their capacity to address specific biological barriers during distinct stages of periodontal healing, as summarized in Figure 6. Multiphasic and gradient scaffolds provide spatially compartmentalized cues that support tissue-specific differentiation and facilitate the re-establishment of the cementum–PDL–bone interface. Spatiotemporally programmable delivery systems coordinate cell recruitment and lineage-specific signaling while limiting epithelial competition during early wound healing. Stimuli-responsive antibacterial materials exploit pathological changes such as acidic pH, excessive ROS and elevated enzymatic activity to activate localized antimicrobial and anti-inflammatory functions, thereby protecting the regenerative niche from biofilm recolonization. Immuno-instructive materials reshape the dysregulated immune microenvironment by regulating macrophages and other innate and adaptive immune cells, promoting inflammation resolution and coupling immune regulation with osteogenic and ligamentogenic responses. Injectable, shape-adaptive hydrogels improve material retention and defect conformity in irregular periodontal lesions, whereas bioelectronic and mechanoresponsive systems convert physiological loading into regenerative signals through mechanotransduction pathways. These mechanisms are therefore complementary rather than independent. Their integration within multifunctional platforms may enable sequential control of infection, inflammation, cell recruitment, tissue specification and functional maturation, ultimately shifting periodontal treatment from passive defect filling toward dynamically coordinated regeneration.

**Figure 6:**
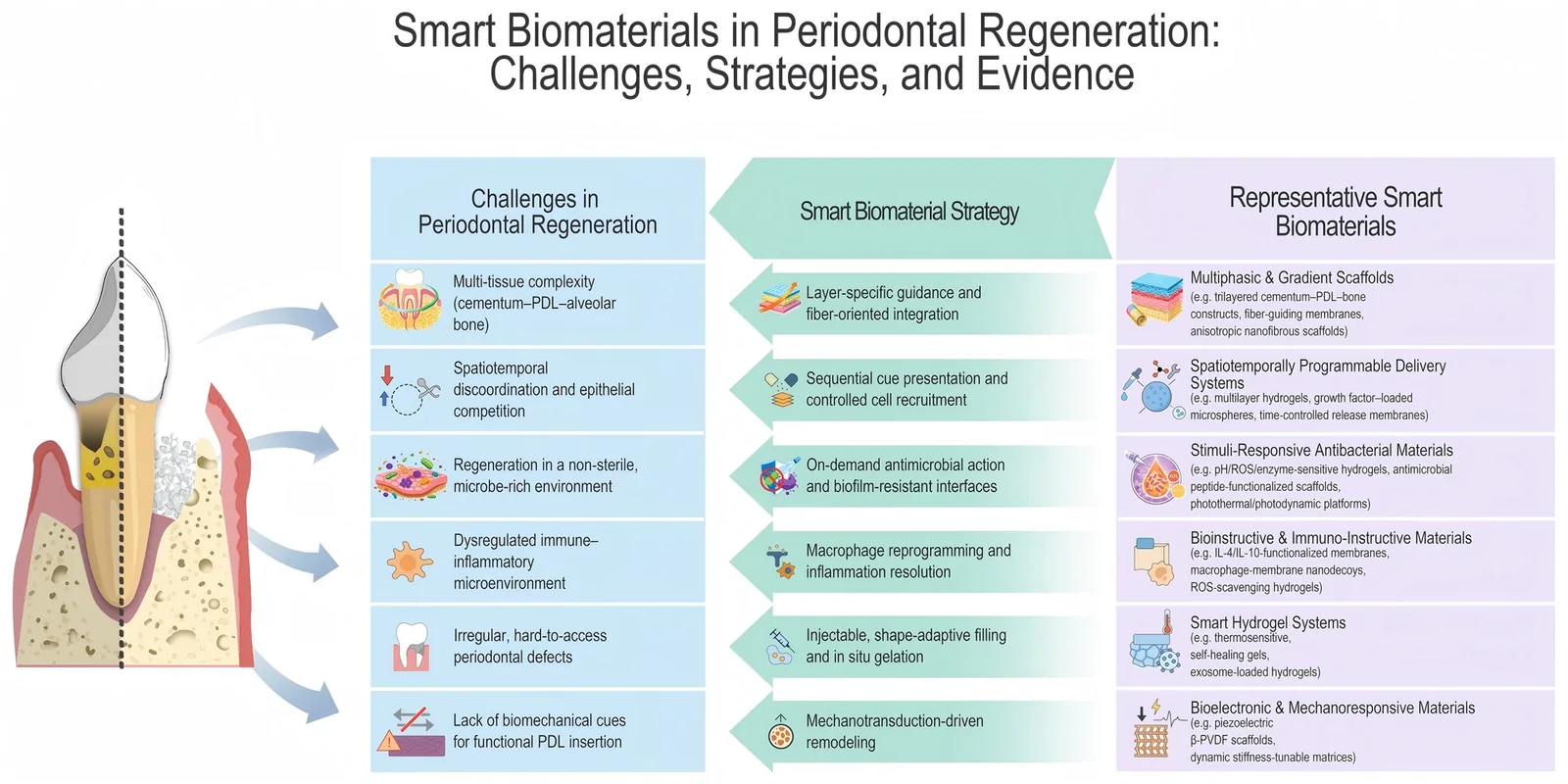
Mapping periodontal regenerative challenges to smart biomaterial strategies and representative systems.

## Preclinical animal studies of smart materials in periodontal regeneration

### Animal models used to evaluate smart materials

A range of animal models has been employed to study smart biomaterials in periodontal regeneration. The selection of appropriate animal models for testing smart biomaterials in periodontal regeneration should be guided by the specific scientific question and the translational stage of the study, as no single model can fully recapitulate human periodontal anatomy, immune responses and disease progression.

#### Rodent models for early-stage screening and mechanistic studies

For early-stage screening and mechanistic studies, rodent models (mice and rats) are recommended. These models are cost-effective, genetically well characterized and allow highly reproducible induction of chronic (e.g. ligature and bacterial inoculation) or acute (fenestration and dehiscence) periodontal defects [29, 72]. They are particularly suitable for evaluating biocompatibility, immunomodulatory effects, angiogenic responses and proof-of-concept regenerative potential of new biomaterials under controlled conditions. In rats, standardized defects with dimensions of approximately 2 × 5 mm are commonly created to enable quantitative evaluation of biomaterial-mediated osteogenesis, early cementum–PDL interface formation and fiber guidance [117–119]. In addition to rat models, mice have been widely used to model periodontal disease and evaluate regenerative strategies. Ligature-induced periodontitis models in mice have been established to investigate gingival inflammation and alveolar bone loss relevant to human periodontitis pathogenesis and regenerative responses [120]. Treatment strategies, such as PLGA-based biomaterial injections, have been shown to reduce inflammation and enhance alveolar bone regeneration in mouse models of periodontitis, supporting their utility for early preclinical evaluation of biomaterial-mediated tissue regeneration [121, 122]. Owing to the limited jaw size, surgical defect models in mice are less standardized in dimension, although small fenestration-type defects (2 × 1.5 mm) have been reported and applied to assess biomaterial- or cell-mediated periodontal regeneration, including bone and PDL responses [123].

#### Rabbit models as an intermediate translational platform

Rabbit periodontal defect models serve as an intermediate translational platform between rodents and large animals, as their larger oral anatomy allows reproducible creation of dehiscence or fenestration defects suitable for multiphasic scaffold implantation and assessment of space maintenance, neovascularization and coordinated cementum–PDL–bone regeneration. However, at commonly used defect sizes (e.g. 4 × 6 mm), these models are mainly applicable for short- to mid-term evaluation (≈ 6 weeks), since continuous tooth eruption and rapid spontaneous healing can obscure long-term stability and functional maturation of periodontal attachment, including Sharpey’s fiber insertion under physiological loading [124]. More recent studies have addressed this limitation by defining a critical-sized mandibular defect in rabbits. Using micro-CT and histological analyses, a box-shaped defect created via an intraoral approach (8 mm × 3 mm × 2 mm) was shown to remain incompletely healed even after 12 weeks, meeting the criteria of a critical-sized defect [125]. This indicates that appropriately sized rabbit mandibular defects can minimize spontaneous healing and enable longer-term evaluation of biomaterial-GTR and integration.

#### Large-animal models for advanced preclinical validation

In contrast, large-animal models (beagle dogs, minipigs and non-human primates) are more appropriate for advanced preclinical validation. These species exhibit periodontal anatomy, defect dimensions, occlusal loading and oral microbiota that more closely resemble the human condition, allowing assessment of spatially coordinated regeneration of cementum, PDL and alveolar bone, as well as surgical handling, space maintenance and long-term functional integration of multiphasic scaffolds and load-responsive materials [20, 73]. In beagle dogs and non-human primates, standardized intrabony defects and furcation defects are most commonly used. In beagle dog models, standardized periodontal intrabony defects are typically created with a vertical depth of approximately 4–6 mm and a mesio–distal width of 4–5 mm [126, 127]. For two- or three-wall defects, the bucco–lingual dimension is also standardized (approximately 3–5 mm), resulting in three-dimensional defects of about 5 × 5 × 5 mm or 6 × 5 × 4 mm, which provide a reproducible and clinically relevant regenerative challenge [128, 129]. For furcation defects classified as Class II or Class III, the vertical and horizontal defect dimensions typically range from 3 to 5 mm [130–132]. These defects involve the complete removal of the alveolar bone, cementum and PDL within the defect area, thereby enabling evaluation of true periodontal regeneration, including new cementum formation, PDL fiber insertion and bone fill.

In minipigs, critical-size one-wall or two-wall intrabony defects (approximately 5–8 mm in depth and 4–6 mm in width) are often created in the premolar–molar region, where the thickness of the alveolar bone and the size of the roots permit implantation of clinically relevant scaffolds and membranes and assessment of space maintenance and vascularization under physiological loading [133–135].

#### Translational considerations for animal model selection

Overall, as summarized in Figure 7 and Table 2, no single animal model can fully recapitulate the complexity of human periodontal regeneration. Instead, a stepwise, question-driven strategy that progresses from rodents to rabbits and subsequently to large-animal models provides a rational framework for evaluating smart biomaterials and improving the translational relevance of preclinical studies. This tiered approach balances experimental feasibility with physiological relevance, enhances translational predictability and provides practical guidance for rational animal selection based on defect type, inflammatory context, mechanical demands and the functional attributes of the smart biomaterial, rather than a one-size-fits-all model.

**Figure 7:**
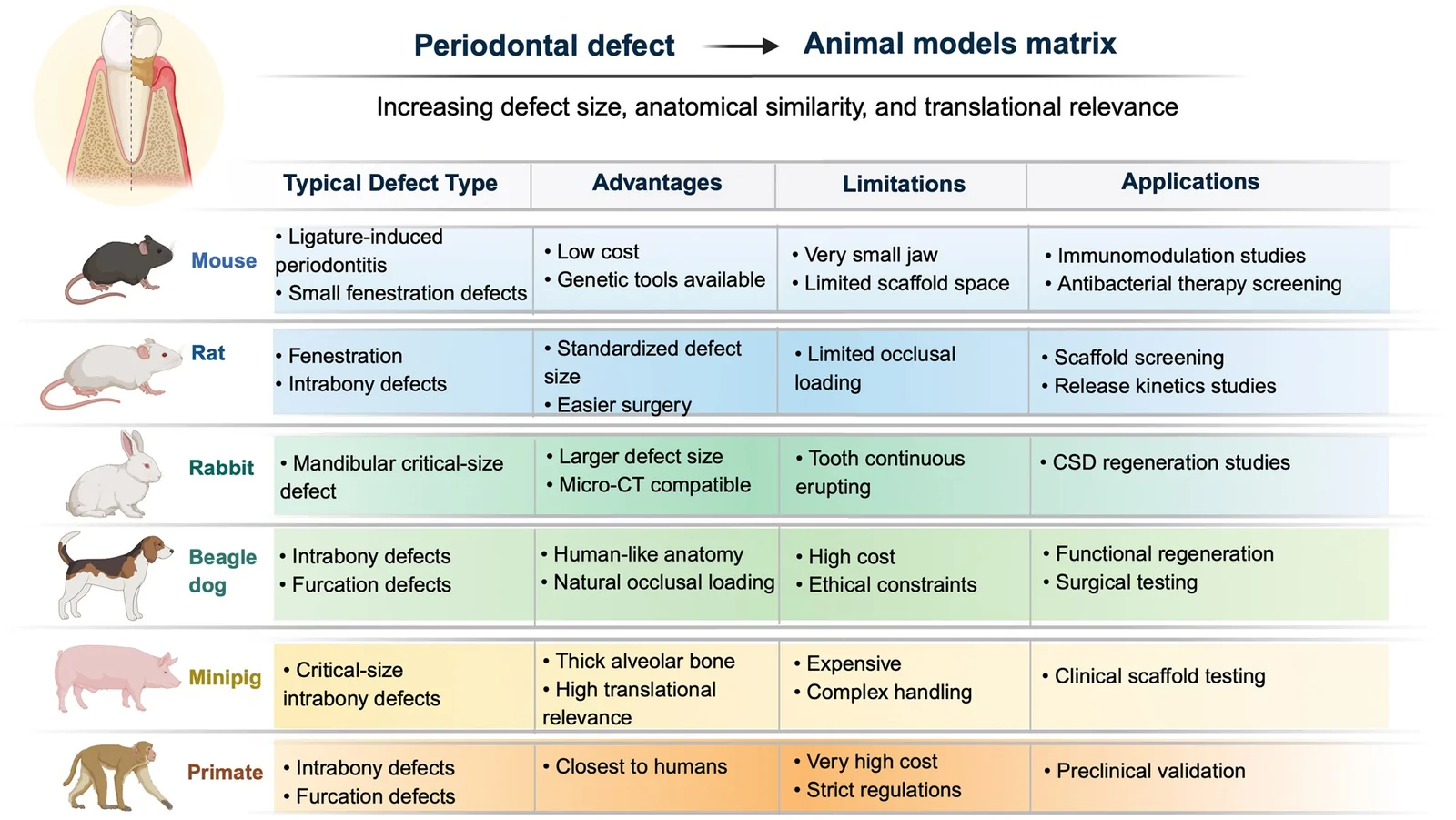
Animal model matrix for periodontal defects.

**Table 2:** Recommended animal models for testing smart biomaterials in periodontal regeneration.

Translational stage	Animal model	Typical defect type	Common defect size	Main evaluation focus	Key advantages	Main limitations	Recommended applications
Early-stage screening & mechanistic studies	Mouse [72, 123, 136]	Ligature-induced periodontitis; small fenestration	Ligature model (bone loss, no fixed size); fenestration ∼ 2 × 1.5 mm	Inflammation, immune modulation, angiogenesis, early osteogenesis, proof-of-concept regeneration	Low cost; genetic tools; reproducible inflammation models	Very small jaw; limited space for scaffolds; mechanical loading not comparable to humans	Immunomodulation, antibacterial effects, growth factor and cell therapy screening
	Rat [117, 118]	Fenestration/dehiscence; intrabony defects	∼ 2 × 5 mm (width × length)	Biocompatibility, osteogenesis, early cementum–PDL interface, fiber guidance	Standardized defect size; easier surgery than mice; quantitative analysis	Limited occlusal loading; anatomy differs from humans	Scaffold screening, release kinetics, PDL orientation
Intermediate structural validation	Rabbit [124, 125]	Fenestration/dehiscence; mandibular critical-size defect (CSD)	Conventional: ∼ 4 × 6 mm (short-term); CSD: 8 × 3 × 2 mm	Space maintenance, vascularization, multiphasic scaffold integration, mid-term regeneration	Larger defect size; accommodates complex scaffolds; micro-CT compatible	Continuous eruption; rapid spontaneous healing (non-CSD models)	Short–mid-term coordinated cementum–PDL–bone regeneration; long-term testing using mandibular CSD
Advanced preclinical validation	Beagle dog	Intrabony (one-/two-wall), furcation (Class II/III)	Intrabony: ∼ 4–6 mm depth × 4–5 mm width [126, 128]; Furcation: ∼ 3–5 mm vertical/horizontal [130, 131]	True periodontal regeneration, Sharpey’s fiber insertion, load-bearing function	Human-like anatomy, occlusal loading, microbiota	High cost; ethical constraints	Functional attachment regeneration; surgical feasibility
	Minipig [133–135]	One-/two-wall intrabony critical-size defects	∼ 5–8 mm depth × 4–6 mm width	Space maintenance, vascularization, long-term scaffold stability	Thick alveolar bone; large roots; translational relevance	Expensive; complex handling	Testing clinically sized multiphasic scaffolds and membranes
	Non-human primate	Intrabony; furcation (Class II)	Intrabony: ∼ 7–9 mm depth × 2–3 mm width [137]; 3–5 mm furcation [132]	Long-term functional regeneration under realistic microbial and mechanical environment	Closest to human periodontium	Very high cost; strict ethics	Final validation before clinical translation

Among currently available models, non-human primates may provide the highest degree of physiological and anatomical similarity to humans [138]; however, their use is severely restricted by ethical considerations, high costs and limited availability. Consequently, large-animal models such as beagle dogs and minipigs are generally regarded as the most clinically relevant options for preclinical evaluation of periodontal regenerative biomaterials [139]. Although both models offer high translational value, each possesses distinct advantages. Minipigs exhibit dentoalveolar anatomy, periodontal tissue architecture and bone-healing characteristics that closely resemble those of humans, making them particularly attractive for evaluating regenerative biomaterials in clinically relevant defect configurations [140]. In contrast, beagle dogs remain the most extensively validated large-animal model in periodontal regeneration research owing to their well-established periodontal defect models, predictable healing responses and long history of use in the evaluation of GTR, growth factors, stem-cell-based therapies and regenerative biomaterials [141]. Consequently, minipigs may offer greater anatomical relevance, whereas beagle dogs currently provide stronger translational evidence and greater comparability with existing periodontal regeneration studies. Therefore, the choice of model should be guided by the specific objectives of the study, and a stepwise translational strategy from rodents to large-animal validation remains the most appropriate pathway toward clinical application.

Nevertheless, even the most widely used models possess inherent limitations. Despite their widespread use, current animal models present important translational limitations. Rodent models provide valuable mechanistic insights but poorly replicate human occlusal loading, periodontal anatomy and microbial ecology. Large-animal models offer superior anatomical relevance, yet high cost, ethical constraints and limited standardization reduce reproducibility across studies. Moreover, substantial variability in defect design, healing duration and outcome assessment complicates cross-study comparison and may contribute to inconsistent conclusions regarding biomaterial efficacy.

## Clinical studies and translational considerations

To date, the clinical translation of smart biomaterials for periodontal regeneration remains in its early stages, with a pronounced gap between extensive preclinical advances and routine clinical application. Among the various material classes, only bioinstructive growth factor-based systems have accumulated relatively robust clinical evidence, supported by multiple randomized controlled trials and histological validation of true periodontal regeneration. In contrast, the clinical evidence for other advanced platforms, including stimuli-responsive, immunomodulatory and bioelectronic/mechanoresponsive materials, remains very limited, with most data restricted to *in vitro* and animal studies (Tables 3–6).

**Table 3:** Clinical studies of stimuli-responsive smart biomaterials for periodontal regeneration.

Biomaterial category	Study design (*n*)	Defect type	Major findings	Key reference
Thermo-responsive hydrogels	RCT (*n* = 40)	Intrabony defects	Significant CAL gain, PD reduction and increased radiographic bone fill, indicating clinical osteogenic and regenerative efficacy	Eshwar *et al.* (2023) [142]
pH-responsive systems	Clinical evaluation is still lacking	—	Shown to enable environment-triggered drug release in vitro and in vivo; no periodontal regeneration trials in humans to date	—
ROS/enzyme-responsive hydrogels	Clinical evaluation is still lacking	—	Preclinical studies indicate inflammation-responsive degradation and on-demand release; clinical translation not yet reported	—
Photo-responsive/photothermal platforms	Clinical evaluation is still lacking	—	Used experimentally for antibacterial and immunomodulatory purposes; no direct evidence for periodontal tissue regeneration in humans	—

**Table 4:** Clinical studies of bioinstructive smart biomaterials for periodontal regeneration.

Biomaterial category	Study design (*n*)	Defect type	Major findings	Key reference
Growth factor–functionalized scaffolds (e.g., rhPDGF-BB + β-TCP; rhFGF-2 + collagen/HA/ABG; EMD-coated matrices)	Multiple randomized controlled clinical trials	Intrabony defects; Class II furcation defects	Significant CAL gain, PD reduction and radiographic bone fill; histologic evidence of true periodontal regeneration (new cementum, PDL and alveolar bone)	See Table 5 for detailed clinical references
Drug-loaded bioactive surfaces (Janus collagen film with diclofenac sodium)	Clinical evaluation is still lacking	—	—	—
Nanosilicate-functionalized PCL nanofibrous membranes	Clinical evaluation is still lacking	—	—	—
Macrophage-polarizing supramolecular hydrogel (8-AG + antioxidants)	Clinical evaluation is still lacking	—	—	—
Macrophage-membrane–coated nanodecoys (LL-37-functionalized)	Clinical evaluation is still lacking	—	—	—
Cytokine-modulating Ca-alginate hydrogels (CpG ODN + mangiferin)	Clinical evaluation is still lacking	—	—	—
ATP-responsive Mg/Zn-MOF scaffolds	Clinical evaluation is still lacking	—	—	—

**Table 5:** Clinical studies of growth factor–functionalized bioinstructive scaffolds in periodontal regeneration.

Biomaterial type	Study design (*n*)	Defect type	Major findings	Key reference
rhFGF-2 + autologous bone grafting (ABG)	RCT (*n* = 66)	Intrabony defects	rhFGF-2 (alone or + ABG) showed greater bone defect filling	Kojima *et al.* (2024) [143]
rhFGF-2 local delivery (carrier/gel-based intraoperative application)	RCT (*n* = 253)	Intrabony defects	Significant clinical attachment gain and radiographic bone fill compared with controls, supporting the regenerative efficacy of rhFGF-2	Kitamura *et al.* (2011) [144]
rhFGF-2 + β-TCP (growth factor–scaffold composite)	RCT (*n* = 88)	Intrabony defects	rhFGF-2 significantly enhanced clinical and radiographic outcomes over β-TCP alone, indicating improved periodontal regeneration	Cochran *et al.* (2016) [145]
rhFGF-2 + hyaluronic acid (HA)	RCT (*n* = 60)	Intrabony defects	Greater probing depth reduction and clinical attachment gain in the rhFGF-2/HA group compared with controls	Santana *et al.* (2015) [146]
rhFGF-2 + deproteinized bovine bone mineral (DBBM)	RCT (*n* = 32)	Intrabony defects	Higher radiographic bone fill and improved CAL in the rhFGF-2–treated sites	Saito *et al.* (2019) [147]
rhPDGF-BB + β-TCP (GEM 21S)	RCT (*n* = 180)	Intrabony defects	Significant CAL gain and bone fill, with accelerated early healing compared with control grafts	Nevins *et al.* (2005) [148]
rhPDGF-BB + bone graft/membrane	Human histologic and clinical study	Class II furcation and intrabony defects	Histologic evidence of true periodontal regeneration with new cementum, PDL and alveolar bone formation	Nevins *et al.* (2003) [149]

**Table 6:** Clinical evidence of bioelectronic and mechanoresponsive materials in periodontal tissue regeneration.

Biomaterial category	Study design (*n*)	Defect type	Major findings	Key reference
Piezoelectric materials (BaTiO₃–chitosan membranes; PVDF/P(VDF-TrFE) nanofibers; KNN composites)	Clinical evaluation is still lacking	—	—	—
Conductive polymers (PPy-coated scaffolds; PEDOT: PSS hydrogels; polyaniline composites)	Clinical evaluation is still lacking	—	—	—
Electroactive 3D scaffolds (conductive polymer–coated 3D-printed PLA)	Clinical evaluation is still lacking	—	—	—
Mechano-topographic scaffolds (aligned/micropatterned fibrous matrices)	Clinical evaluation is still lacking	—	—	—
Mechanically tuned hydrogels (stiffness- and viscoelasticity-programmed hydrogels)	Clinical evaluation is still lacking	—	—	—

Within currently available smart biomaterial platforms, the level of clinical evidence remains highly uneven. Among stimuli-responsive systems, thermo-responsive hydrogels represent the most advanced category, with at least one randomized clinical trial demonstrating significant improvements in clinical attachment gain, probing depth reduction and radiographic bone fill in intrabony defects [142]. More robust clinical evidence exists for growth factor-functionalized scaffolds, including rhFGF-2, rhFGF-2/β-TCP composites and rhPDGF-BB-based regenerative systems, which have consistently demonstrated significant clinical improvements and, in some cases, histological evidence of true periodontal regeneration in human intrabony and furcation defects [143, 145–148]. By contrast, most other emerging platforms, including pH-responsive, ROS-responsive, immunomodulatory and bioelectronic/mechanoresponsive systems, remain supported primarily by *in vitro* and animal studies, with no dedicated human periodontal regenerative trials reported to date.

Despite encouraging preclinical results, the clinical translation of these advanced systems remains significantly slower than their experimental development. Major translational barriers include the limited predictive value of small-animal models, challenges in scalable and reproducible manufacturing, sterilization compatibility, long-term biosafety assessment, regulatory approval for multifunctional bioactive components and the lack of standardized clinical endpoints across studies [150]. Furthermore, the heterogeneity of periodontal defect morphology, microbial burden, immune status and functional loading conditions in human patients add additional complexity to clinical implementation. These challenges highlight the need to clearly distinguish experimental promises from clinically validated therapeutic strategies and to establish more rigorous translational pathways for future smart biomaterial development.

Overall, these findings highlight a substantial translational gap between mechanistic innovation and clinical implementation in periodontal regenerative biomaterials. Future studies should prioritize standardized clinical endpoints, long-term safety evaluation, scalable manufacturing and well-designed multicenter clinical trials. In addition, integrating patient-specific biological, microbial and biomechanical factors into biomaterial design may further accelerate the clinical translation of next-generation smart regenerative systems.

## Recent progress in clinical translation

Recent advances in smart biomaterials for periodontal regeneration have yielded several representative showcases in bioactivity, structure and stimuli-responsiveness in achieving targeted tissue regeneration.

Modular biphasic scaffolds offer spatial and functional compartmentalization that mimics the native periodontium. Wei *et al.* [151] developed a biphasic scaffold that combined a mineralized lamellar bone-mimetic phase with a fibrous ligamentous phase, enabling region-specific support for alveolar bone and PDL regeneration. The system facilitated collagen fiber orientation, cementum-like tissue formation and continuous mineralized-ligamentous integration in a rat model, illustrating the potential of architectural modularity in achieving complex tissue regeneration.

TRC-mimetic (triple-region composition) titanium nanosurfaces represent another innovation, engineered to mimic the heterogeneous architecture of the periodontium [152, 153]. Researchers designed nanosurfaces with region-specific topographies and biochemical modifications that separately promoted osteogenesis, cementogenesis and ligamentogenesis [154]. *In vitro* and *in vivo* studies confirmed the capacity of these TRC-nanosurfaces to guide PDL orientation, enhance cementum deposition and achieve functional integration between titanium implants and periodontal tissues.

Thermosensitive hydrogels with bioactive payloads exemplify a minimally invasive strategy for regenerative delivery. Amiri *et al.* (2024) [20] reported the application of injectable, thermoresponsive hydrogels incorporating anti-inflammatory and osteogenic agents in a beagle periodontal defect model. These hydrogels exhibited sol–gel transitions at physiological temperature and released their payloads in a sustained manner. Histological evaluation showed significant improvement in new bone and PDL formation, with a reduction in inflammatory infiltration and enhanced cementum regeneration at the root surface.

Together, these representative studies reflect a paradigm shift from passive scaffolds to dynamic, bioactive systems capable of sensing, responding and instructing regeneration. These findings support the potential value of integrating material science, immunology and bioengineering to resolve the complexity of periodontal regeneration.

## Advantages and remaining challenges for smart biomaterials in periodontal regeneration

Smart biomaterials have introduced a paradigm shift in periodontal regeneration by enabling materials to actively sense, respond to and modulate the pathological microenvironment rather than serving as passive space fillers. Their major advantages lie in their ability to provide spatiotemporal control of bioactivity, adapt to patient-specific defect morphologies and loading conditions, coordinate the regeneration of multiple periodontal tissues and actively regulate the local immune–inflammatory milieu. Despite rapid technological advances, the translation of these advances into predictable, routine clinical therapies remains limited. Most smart platforms remain limited to *in vitro* and small-animal studies, with only a few advancing to large-animal validation or well-designed clinical trials. This translational gap reflects persistent challenges related to manufacturing scalability, regulatory approval and long-term biological safety and efficacy.

### Advantages

#### Spatiotemporal control of therapeutic cues

Smart biomaterials enable precise spatiotemporal control of therapeutic signals, particularly through stimuli-responsive systems that release bioactive molecules in response to pathological microenvironmental cues, such as acidic pH, elevated reactive oxygen species, ATP release or increased protease activity. This on-demand, site-specific activation allows therapeutic agents to be preferentially delivered to inflamed or actively remodeling periodontal tissues, thereby enhancing local efficacy while minimizing off-target exposure and systemic side effects. For example, ATP-responsive scaffolds and pH-sensitive hydrogels have been shown to achieve inflammation-triggered drug release, synchronizing therapeutic action with the dynamic phases of periodontal wound healing and extracellular matrix remodeling [72].

#### Patient-specific adaptability and minimally invasive delivery

Injectable and *in situ*-gelling systems enable smart biomaterials to conform to highly irregular periodontal defects and complex root surface geometries, addressing a major limitation of preformed membranes or blocks. Thermosensitive hydrogels, such as those reported by Amiri *et al.* (2024) [20], undergo sol–gel transition at body temperature, facilitating flapless or minimally invasive delivery and intimate defect filling. In parallel, mechanoresponsive and piezoelectric scaffolds (e.g. PVDF-based systems) can adapt to physiological occlusal loading, providing dynamic mechanical and electrical cues that better mimic the native periodontal niche [155].

#### Multi-tissue and interface-oriented regeneration

The periodontium is a hierarchically organized, multi-tissue complex comprising cementum, periodontal ligament and alveolar bone. Smart biomaterials with modular, gradient or Janus architectures are uniquely suited to recapitulate this spatial heterogeneity. Dual-layer electrospun membranes, Janus scaffolds and supramolecular self-assembling hydrogels can present distinct biochemical and mechanical cues on opposing surfaces, thereby simultaneously supporting osteogenesis, ligament fiber alignment and cementogenic differentiation [72, 156]. Such spatial programming is critical for restoring functional attachment rather than merely achieving bone fill.

#### Active immunomodulation and inflammation resolution

Beyond structural and biochemical guidance, immuno-instructive smart materials can actively reprogram the host immune response. By promoting macrophage polarization toward a pro-regenerative M2 phenotype and suppressing excessive pro-inflammatory cytokines (e.g. TNF-α and IL-1β), these systems help terminate chronic inflammation and establish a regenerative microenvironment [157]. Macrophage membrane–camouflaged nanodecoys, immune-responsive Janus scaffolds and cytokine-modulating hydrogels have demonstrated the capacity to couple immune regulation with enhanced periodontal tissue formation [59], highlighting immune modulation as a central mechanism linking material design to functional regeneration.

### Remaining challenges

Although smart biomaterials have demonstrated promising regenerative potential in preclinical studies, several unresolved challenges remain before their clinical applicability can be established. An overview of these challenges is presented in Figure 8.

**Figure 8:**
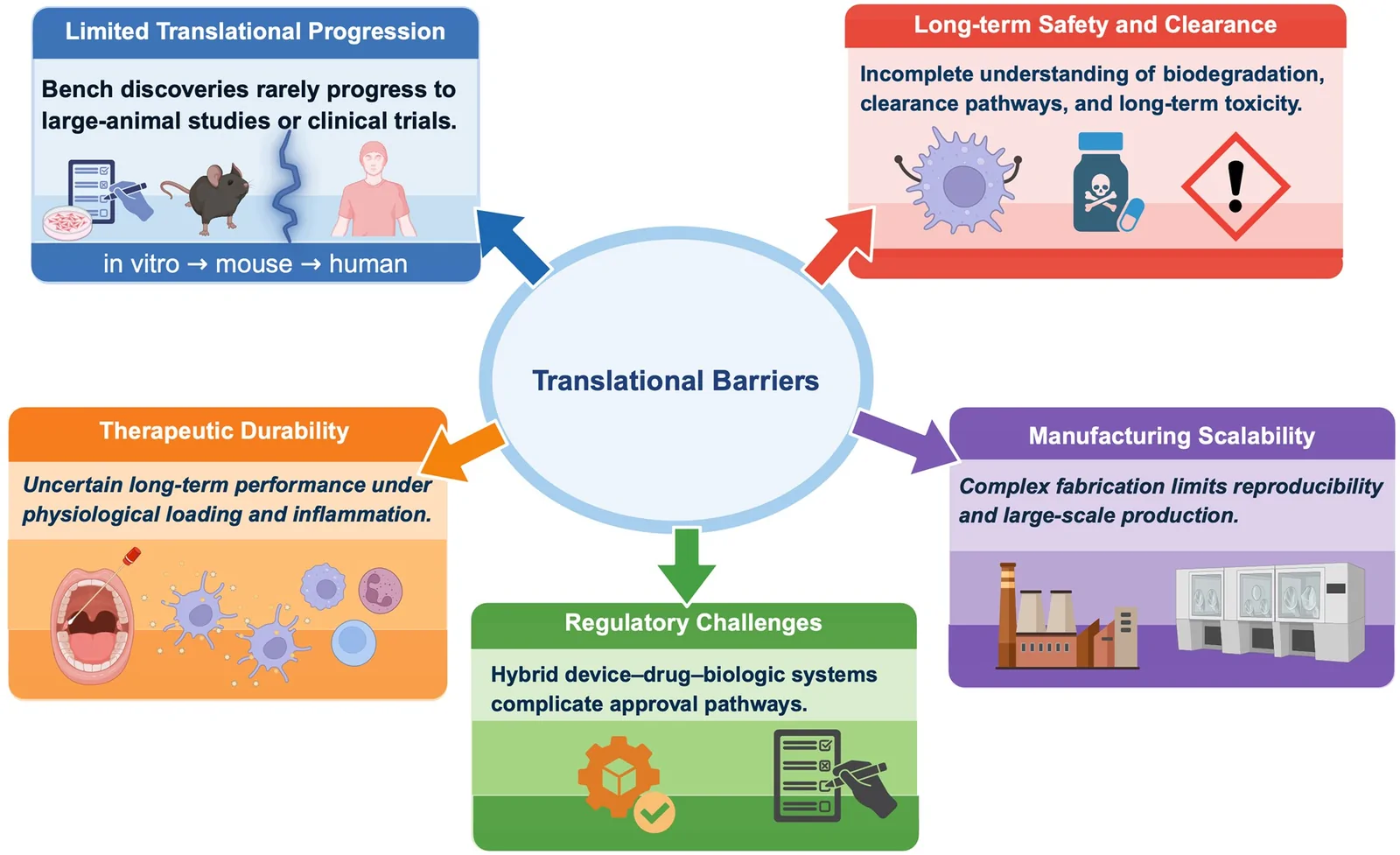
Translational barriers of smart biomaterials for periodontal regeneration.

#### Insufficient translational progression and clinical-level validation

Despite impressive laboratory advances, most smart platforms remain confined to *in vitro* studies and small-animal models, with only a limited number progressing to large-animal validation and even fewer entering rigorously designed clinical trials. A substantial proportion of resources is still concentrated at the material design and proof-of-concept stage, while systematic translational evaluation, covering scalable manufacturing, standardized quality control, long-term safety and functional durability, remains insufficiently completed. This incomplete developmental pipeline represents a major bottleneck, limiting the progress of many promising biomaterials platforms from preclinical validation toward clinical evaluation.

#### Long-term *in vivo* safety, biocompatibility and clearance

The long-term *in vivo* fate, biodegradation and clearance pathways of novel nanomaterials, electrically active fillers, and their degradation products remain insufficiently characterized. Particular attention should be paid to smart biomaterials containing inorganic nanoparticles, piezoelectric ceramics, magnetic particles or metal-based components, as the long-term consequences of ion release, nanoparticle accumulation and degradation products remain incompletely understood. For example, materials incorporating barium-, cobalt- or other metal-containing constituents may require comprehensive evaluation of biodistribution, immunocompatibility, systemic toxicity and clearance before clinical translation. Potential risks, including chronic inflammation, immune sensitization, fibrotic encapsulation and off-target bioelectrical effects, highlight the need for systematic and long-term evaluation of biocompatibility, toxicology, functional safety and material elimination routes before these smart platforms can be safely translated into clinical practice [73].

#### Long-term therapeutic durability and functional integration

While many smart systems show impressive short-term regenerative outcomes in preclinical models, their long-term performance under physiological mechanical loading and inflammatory challenges is largely unknown. Critical issues include maintenance of biomechanical integrity, stable integration with host vasculature and neural networks and avoidance of fibrous encapsulation or late immune dysregulation. Furthermore, inter-patient variability in immune status, defect geometry and occlusal forces underscores the need for adaptive or feedback-controlled systems capable of responding to evolving biological and mechanical conditions [29, 59].

Closely related to these long-term challenges is the issue of biomechanical compatibility throughout the regenerative process. Unlike conventional bone substitutes that primarily provide structural replacement, many smart biomaterials act as temporary regenerative templates that guide endogenous tissue formation. Nevertheless, these materials must still provide sufficient mechanical support while allowing physiological load transfer to newly forming tissues. Given the marked mechanical heterogeneity of the bone–PDL–cementum complex, future biomaterial designs may require spatially controlled stiffness gradients and dynamic mechanical adaptability to better support functional periodontal attachment and long-term tissue stability.

#### Manufacturing complexity and scalability

The sophisticated architectures and multifunctional components that confer responsiveness also make fabrication technically demanding. Multistep functionalization, precise control of spatial gradients, incorporation of labile biologics and stringent requirements for sterilization, storage and transportation pose substantial challenges to large-scale, cost-effective production and batch-to-batch reproducibility. Platforms such as Janus membranes, supramolecular hydrogels and piezoelectric composites often rely on tightly controlled processing conditions, which further complicate industrial scale-up and clinical deployment [29, 79].

#### Regulatory complexity and approval pathways

Smart biomaterials often integrate characteristics of medical devices, drug delivery systems and biologics within a single construct [20, 158]. This multifunctionality blurs traditional regulatory boundaries and results in complex and prolonged approval pathways, as such platforms may need to simultaneously satisfy the requirements of multiple regulatory frameworks.

## Future directions and emerging trends

The evolution of smart biomaterials in periodontal regeneration is increasingly intertwined with cutting-edge technologies such as 4D printing, omics-guided personalization and intelligent immunoengineering. These trends suggest a shift from single-functional to multiresponsive, bioadaptive and patient-tailored platforms.

### Four-dimensional printing and biofabrication: adaptive reconstruction of irregular defects

Four-dimensional printing represents a transformative approach that introduces temporal shape-shifting capabilities to biomaterials. Unlike static scaffolds, 4D printed smart constructs can dynamically respond to local microenvironmental cues such as pH or enzyme levels, adjusting drug release or scaffold conformation accordingly [159, 160]. For instance, thermo-pH dual-responsive hydrogels have demonstrated dynamic structural adaptability and drug release modulation suitable for periodontal pockets, paving the way for deployable printed systems that conform to irregular defect geometries [161].

Recent progress in bioink formulation and scaffold microarchitecture allows layered integration of growth factors and cell-homing signals, accelerating site-specific tissue repair [162]. Hybrid systems integrating bioceramics and stimuli-responsive polymers are being explored for customized biofabricated interfaces between bone, cementum and ligament layers [151].

### Multiresponsive and hybrid systems: spatiotemporal control in a multifactorial inflammatory and infectious microenvironment

To address the complexity of periodontal lesions, multi-responsive systems combining redox-, enzyme-, pH- and temperature sensitivity have emerged [163]. These hybrid scaffolds enable temporally staged drug release, antibacterial action and immune reprogramming. A notable example is a CuS-loaded hydrogel that demonstrated antibacterial efficacy, promoted stem cell proliferation and enhanced periodontal regeneration *in vivo* through self-healing and enzyme-sensitive release mechanisms [164].

Likewise, a triple-crosslinked PNIPAM-chitosan hydrogel incorporating silver-lipid complexes achieved sustained antibacterial function, inflammation resolution and bone regeneration through ROS-triggered and thermal stimuli [165]. These systems are anticipated to converge with sensor-driven feedback loops in future ‘intelligent’ periodontal devices.

### Omics- and AI-guided smart scaffolds: precision and patient-specific regeneration

The integration of omics technologies and artificial intelligence (AI) analytics can guide the design of personalized biomaterials by aligning scaffold properties with patient-specific transcriptomic or microbiome signatures. For example, scRNA-seq analysis has elucidated mechanoresponsive fibroblast subsets within the periodontal ligament that respond to distinct loading stimuli [98], which could inform scaffold patterning and stiffness tuning at the microscale.

AI-based modeling platforms have already been employed to optimize scaffold porosity, degradation rates and delivery profiles. These tools could synergize with intraoral imaging and 3D printing to deliver personalized, defect-matching constructs embedded with individually dosed biologics.

Beyond patient stratification, AI and machine learning (ML) offer powerful tools for the rational design and optimization of smart biomaterials [166, 167]. Given the complexity of periodontal regeneration, AI-driven approaches can integrate material composition, scaffold architecture, mechanical properties, degradation behavior and biological responses to identify optimal design parameters beyond conventional trial-and-error methods [168, 169]. In the future, combining AI-assisted biomaterial design with patient-specific imaging, omics data and advanced manufacturing technologies may enable the development of personalized smart scaffolds tailored to individual defect characteristics and biological conditions [168].

### Immuno-engineering and antimicrobial regulation: immune and microbiome modulation for a pro-regenerative niche

Beyond tissue regeneration, modulating the local immune milieu and oral microbiome has become central to preventing disease recurrence [170, 171]. Smart biomaterials can now encode cues that promote macrophage polarization toward regenerative M2 phenotypes or release immunosuppressive agents upon detecting high ROS or cytokine levels [23]. Hydrogel systems activating PINK1/Parkin-dependent mitophagy in inflammatory macrophages effectively reduced oxidative damage and sustained M2 dominance, leading to enhanced alveolar bone regeneration [172].

Future scaffolds may integrate microbiota-targeted peptides or prebiotics, enabling simultaneous suppression of keystone pathogens (e.g. *P. gingivalis*) and enhancement of commensal flora, thereby reconstructing a symbiotic periodontal niche.

### Exosome-loaded and decellularized smart matrices: clinically translatable cell-free regenerative platforms

Cell-free therapies using engineered exosomes, extracellular vesicles (EVs) and decellularized matrices offer immunologically safe, scalable alternatives to cell transplantation. Injectable hydrogels incorporating EVs from mesenchymal stem cells or macrophages have demonstrated the capacity to enhance cementogenesis, collagen alignment and angiogenesis in rat and canine models [173].

Similarly, decellularized ECM constructs embedded within pH- or enzyme-responsive matrices provide structural cues and release bioactive fragments that activate endogenous progenitor cells [174]. These systems may provide a promising foundation for future GMP-compliant biomanufacturing and translational evaluation.

In addition, future development of smart biomaterials should extend beyond healthy experimental models and address the challenges associated with severe periodontal defects and medically compromised patients. Conditions such as diabetes, aging, smoking and immune dysregulation are characterized by persistent inflammation, impaired angiogenesis, oxidative stress and reduced regenerative capacity, which may compromise treatment outcomes [175, 176]. In these settings, multifunctional smart biomaterials capable of simultaneously modulating inflammation, scavenging excessive ROS, promoting angiogenesis and delivering regenerative cues may offer significant advantages over conventional materials. Furthermore, patient-specific biomaterial designs guided by disease status, inflammatory profiles and regenerative potential may improve therapeutic predictability in complex clinical scenarios.

## Conclusion

Smart biomaterials represent a paradigm shift in periodontal regeneration, evolving from passive scaffolds into dynamic, immuno-instructive therapeutic systems. Through stimuli responsiveness, multifunctional integration and spatiotemporal control, they enable coordinated regeneration within complex periodontal tissues. Continued interdisciplinary collaboration and translational standardization will be essential to facilitate the responsible clinical translation of smart biomaterials in precision and personalized periodontics. Smart materials are not merely tools but active biological participants in periodontal healing. Their evolution marks a decisive step toward precision periodontics and opens new frontiers in minimally invasive, adaptive and personalized tissue regeneration.

## Supplementary Material

rbag182_Supplementary_Data
