# Supplementary material for: Reimagining periodontal regeneration: smart biomaterials for a complex tissue landscape
Source: Regen Biomater. 2026 Aug 20;13:rbag182. doi: 10.1093/rb/rbag182 (PMC13633685; doi:10.1093/rb/rbag182)
Supplement: Supplementary file 1 [file rbag182_supplementary_data.docx]

Table S1. Comparative biomechanical characteristics and compatibility of smart biomaterial systems for periodontal regeneration.

| **Biomaterial system** | **Relative stiffness (vs native alveolar bone)** | **Elastic modulus** | **Compressive modulus** | **Tensile modulus** | **Viscoelasticity** | **Load-bearing capability** | **Mechanical compatibility with native alveolar bone** |
| --- | --- | --- | --- | --- | --- | --- | --- |
| **Native alveolar bone (reference)** | **High** | **High (15–20 GPa)[1, 2]** | **High** | **Moderate** | **Low** | **High** | Native load-bearing tissue |
| pH-/Redox-responsive hydrogels | Very low | Very low | Low | Very low | High | Poor | Suitable for defect filling but insufficient structural support |
| Thermosensitive hydrogels | Very low | Very low | Low | Very low | High | Poor | Excellent conformity but poor space maintenance |
| Enzyme-responsive biomaterials | Low | Low | Low–Moderate | Low | High | Poor–Moderate | Dynamic remodeling but limited mechanical strength |
| Bioinstructive scaffolds | Moderate | Moderate | Moderate–High | Moderate | Moderate | Moderate–High | Provide structural guidance and partial mechanical support |
| Immunomodulatory hydrogels | Very low | Very low | Low | Very low | High | Poor | Primarily designed for biological regulation rather than load-bearing |
| Piezoelectric membranes/scaffolds | Moderate–High | Moderate–High | High | Moderate | Low–Moderate | High | Better force transmission and mechanotransduction, but may exceed native tissue stiffness |
| Conductive polymers | Moderate | Moderate | Moderate | Moderate | Low–Moderate | Moderate | Mechanical properties highly formulation-dependent |
| Piezoelectric nanocomposite hydrogels | Low–Moderate | Low–Moderate | Moderate | Low–Moderate | Moderate–High | Moderate | Improved mechanical stability while retaining hydrogel adaptability |

**Note:** Mechanical properties are presented qualitatively because they vary considerably among material formulations and fabrication methods. The comparison is intended to illustrate general biomechanical compatibility with the native alveolar bone environment rather than provide absolute material parameters.

**References**

[1]	Lakatos É, Magyar L, Bojtár I. Material Properties of the Mandibular Trabecular Bone. Journal of Medical Engineering. 2014;2014(1):470539.

[2]	Seong WJ, Kim UK, Swift JQ, Heo YC, Hodges JS, Ko CC. Elastic properties and apparent density of human edentulous maxilla and mandible. Int J Oral Maxillofac Surg. 2009;38(10):1088-93.
